# The Evaluation and Treatment of Merkel Cell Carcinoma and Brain Metastasis: A Case Report and Review of the Literature

**DOI:** 10.7759/cureus.51295

**Published:** 2023-12-29

**Authors:** Petr Gaburak, Taylor A Brown, Alexander J Pursel, Luis Cardenas Contreras, Michael Chun

**Affiliations:** 1 Department of Orthopedic Surgery, Elson S. Floyd College of Medicine, Spokane, USA; 2 College of Medicine, Elson S. Floyd College of Medicine, Spokane, USA; 3 Department of Neurology, The Everett Clinic, Everett, USA

**Keywords:** chemoradiation, immunotherapy, neurometastatic, brain metastasis, merkel cell carcinoma, oncology

## Abstract

Merkel cell carcinoma (MCC) is a rare and aggressive neuroendocrine tumor associated with high mortality if metastases are identified. Currently, there is no standardized nor curative treatment for neurometastatic MCC. In this study, we have reviewed the more recent cases and the use of immunotherapy in a population. In this case report and review, we present a case of MCC with brain metastasis currently undergoing treatment with immunotherapy (pembrolizumab) resulting in an initial complete response with a progression-free survival time of five months. We also review the past reported literature and the 11 newly presented cases on their clinical presentation of neurometastatic MCC, immunohistochemical markers, and treatment outcomes. In summary, immunotherapy initially showed a promising response with the complete elimination of MCC brain metastasis. The early aggressive treatment of pembrolizumab with stereotactic radiosurgery should be considered as this treatment plan has shown improved therapeutic effects compared to the standard chemoradiation therapy. Further investigations are needed to determine the efficacy and response of immunotherapy use for neurometastatic MCC.

## Introduction

Merkel cell carcinoma (MCC) is a rare and aggressive cutaneous neuroendocrine tumor that has an incidence of 0.1-1.6 cases per 100,000 people per year and is often associated with high rates of local recurrences and distant metastasis [1,2]. Reichgelt and Visser found the local and distant disease's five-year survival rate as 52% and 17%, respectively [3]. It has been shown that MCC commonly affects middle-aged and elderly individuals and immunocompromised populations [4].

Merkel cells are neuroendocrine-derived and found in the basilar layer of the epidermis. Functionally, Merkel cells are slow-adapting mechanoreceptors that transduce light touch but have been found to have other roles in the endocrine and immune systems [5]. The tumor was first described by Tang and Toker in 1972 as trabecular carcinoma but was later renamed MCC due to the resemblance [6]. The origin has been widely debated since Merkel cells are found to be terminally differentiated and do not have the ability to replicate [7]. However, there are two prevailing theories that propose the origin to be either neural crest-derived or formed within the epidermis [8]. The pathogenesis of MCC has been associated with increased ultraviolet (UV) radiation exposure, immunosuppression (shown in several studies of patients with HIV), and the more recent finding of the Merkel cell polyomavirus (MCPyV) [9-11].

Classically, MCC presents with a primary cutaneous lesion in sun-exposed regions of the skin as a painless erythematous nodule with the potential to ulcerate and grow rapidly within a short period of time. The metastasis of MCC is often lymphatic with half of the patients presenting with positive lymph nodes at diagnosis [12]. Common sites of metastasis are regional lymph nodes, the liver, the lung, the bone, and rarely the brain [2].

Due to uncommon central nervous system (CNS) involvement, detection is often delayed, and treatment protocols are not well established. Previous studies on MCC and CNS metastasis have analyzed diagnosis characteristics and treatment outcomes of chemoradiation therapy; in this study, we aim to determine the following: is there preliminary evidence for the use of immunotherapy in this specific population?

We identified 40 prior cases of the neurometastatic involvement of MCC in the literature with 10 new cases reported in the last five years. Here, we also report a case of a patient with MCC involvement in the brain presented at our institution in 2021. With the addition of the 11 newly presented cases to the previously reported series, we aim to review the characteristics involved in the evaluation of MCC with metastasis to the brain and treatment protocols that include immunotherapy.

## Case presentation

The case report and literature review were developed and presented in accordance with the CARE checklist and guidelines [13]. We retrieved the articles for the literature review using the PubMed search engine with the search terms "neurometastatic MCC" or "neurometastatic merkel cell carcinoma" or "brain and merkel cell carcinoma" or "neurometastatic and MCC" or "Brain AND MCC." Articles identified and collected were case reports, case series, and literature reviews from 1980 to the present day.

An 87-year-old Caucasian male with a history of hypertension presented to his internal medicine doctor with a subcutaneous mass that had been present for two weeks on the left posterior proximal upper arm. An ultrasound was performed, and a 2.4 × 1.5 × 2.8 cm hematoma was diagnosed. Three months later, the patient returned to discuss that mass as it had not yet resolved. The doctor ordered an MRI; however, the patient did not get the MRI until one year later when the mass had begun visibly enlarging.

The MRI showed an abnormal mass (2.4 × 3.0 × 3.3 cm) consistent with a neoplasm, as well as abnormally enlarged nearby axillary lymph nodes. The mass and lymph node were biopsied, and a diagnosis of MCC was made; the treatment of the patient's MCC is presented in this case report. The histological staining of the tumor was positive for AE1/AE3 and cytokeratin (CK) 20 and negative for CK7. The lymph node was positive for AE1/AE3, CK20, and synaptophysin and negative for CK7, thyroid transcription factor 1 (TTF-1), and chromogranin.

**Figure 1:**
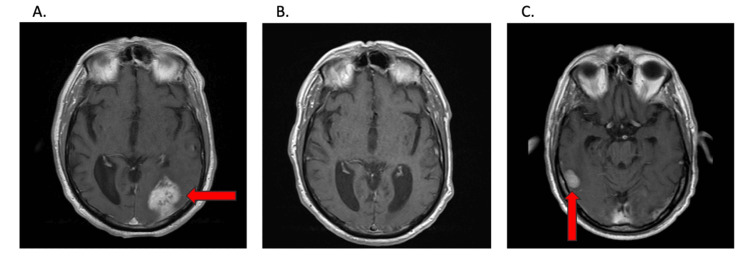
Axial T1-weighted MRI with contrast. (A) Hyperdense in the left occipito-temporal lobe marked with an arrow. (B) The reduction of the left occipito-temporal mass after initiating immunotherapy. (C) New MCC met in the right temporal lobe five months after starting treatment marked with an arrow. MCC: Merkel cell carcinoma

The patient underwent six weeks of radiation therapy (RT), 5,000 cGy, of the subcutaneous mass on the left arm and axillary lymph nodes. Nine months following the completion of radiation therapy, the patient began experiencing word-finding difficulty and aphasia. A brain MRI showed that the patient had a 4.3 cm left temporal mass, and he underwent stereotactic radiosurgery in five fractions (Figure 1A). Due to the patient's age, he was not a candidate for chemotherapy (CH).

Following completion, the patient was started on IV pembrolizumab 200 mg every 3-6 weeks. An MRI done three months following the initiation of pembrolizumab showed no evidence of metastases (Figure 1B). Two months later, the patient experienced blurred vision in his left eye. Imaging showed central retinal artery occlusion and a right temporal mass. A brain MRI showed the mass to be 2.4 × 1.6 × 1.3 cm, and shortly after, stereotactic radiosurgery was performed on the right temporal mass, 3,000 cGy in five fractions (Figure 1C). He tolerated treatment reasonably well without significant adverse effects. The pembrolizumab did cause mild dermatitis that was managed with topical hydrocortisone ointment.

Another brain MRI done four months later showed three new bifrontal metastases. The plan is to perform stereotactic radiosurgery, 2,000 cGy in a single fraction, on these three new lesions and continue pembrolizumab.

## Discussion

**Figure 2:**
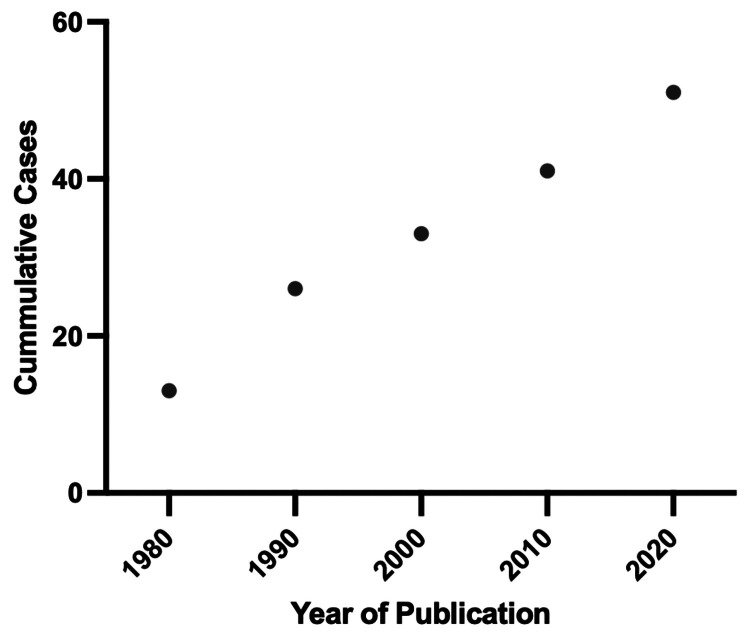
Trend of 51 reported neurometastatic MCC cases over decades. MCC: Merkel cell carcinoma

The incidence of MCC in the United States has increased by 95% between 2000 and 2013, and by 2025, the number of cases is projected to exceed 3,000 [14]. This rise has been speculated to be caused by an increase in the awareness and recognition of the condition but is also attributed to generational differences with respect to prolonged UV exposure. In our review, we provide an analysis of 11 new cases in the last five years showing a 28% increase in reported neurometastatic cases of MCC compared to the previously reported data (Figure 2). The average age at MCC diagnosis was found to be 64 years with a 1.5:1 male-to-female ratio showing slight male predominance (Table 1).

**Table 1:** Literature review findings. SR, surgical resection; RT, radiation therapy; CH, chemotherapy; HT, hyperthermia; G, Gliadel; SRS, stereotactic radiosurgery, IM, immunotherapy; M, male; F, female; R, right; L, left; CNS, central nervous system; Dx, diagnosis

Publication	Age/Sex	Primary Lesion	Location of Metastases in the Brain	Treatment Prior to CNS Disease	Treatment for CNS Metastasis	Time to CNS Disease After Primary Dx	Survival After CNS Metastasis	Overall Survival
Kroll and Toker [15]	48/M	--	Brain	--	SR + RT + CH	--	--	--
	70/M	--	Brain	--	SR + RT + CH	--	--	--
	72/F	--	Meninges	--	SR + RT + CH	--	--	--
Wick et al. [16]	62/M	Face	Brain	SR + RT + CH	SR + RT + CH	12 Months	1 Month	13 Months
	76/M	R Retroarticular Area	Brain	SR		36 Months	0 Months	36 Months
Goepfert et al. [17]	--	--	Brain	--		--	--	--
Giannone et al. [18]	57/F	Scalp	R Frontoparietal	SR	RT + CH	2 Months	7.5 Months	9.5 Months
Grosh et al. [19]	56/M	R Forearm + L Naris + Posterior Neck	Intracerebral + Cerebellar		RT + CH	21 Months	1.5 Months	--
Hitchcock et al. [20]	52/M	L Breast	Cerebral	SR	RT + CH	3 Months	9 Months	12 Months
Knox and Kapp [21]	75/F	R Neck	L Cerebellum	SR + RT + HT	RT	48 Months	50 Months	--
	60/M	L Inguinal Area	Leptomeninges	SR + RT + CH + HT	--	--	1 Month	24 Months
Dudley and Moinuddin [22]	64/M	Nose	Leptomeninges	SR + RT + CH		17 Months	6 Days	17 Months
Alexander et al. [23]	56/M	L Preauricular Area	R Parietal	--	RT + CH	Present at Dx	36 Months	--
Wojak and Murali [24]	78/M	R Parieto-Occipital Area	Calvaria + Dura		SR + RT	Present at Dx	≥12 Months	≥ 12 Months
Manome et al. [25]	57/F	Nasal Cavity	L Frontal Base	SR + RT	SR	Present at Dx	--	--
Small et al. [26]	56/F	L Preauricular Area	Parietal Lobe + Choroid	--	RT + I-125 Episcleral Plaque	Present at Dx	28 Months to Remission	--
Yiengpruksawan et al. [27]	--	--	Brain	--	--	--	--	--
Sharma et al. [28]	57/M	Mid-Lumbar Spine	Cerebral	--	RT + CH	Present at Dx	13 Months	13 Months
Snodgrass et al. [29]	61/M	Forehead	R Parietal + Leptomeninges	SR + CH	RT + CH	12 Months	6 Months	18 Months
Eftekhari et al. [30]	--	--	Brain	--	--	--	--	--
	--	--	Brain	--	--	--	--	--
	--	--	Brain	--	--	--	--	--
Matula et al. [31]	47/M	--	Anterior Skull Base + Frontal Lobe + Dura	--	SR + RT + CH	--	12 Months	12 Months
Straka and Straka [32]	71/F	R Neck	Brain	SR + RT	CH	10 Months	2 Months	12 Months
Litofsky et al. [33]	86/F	R External Auditory Canal	R Temporoparietal Lobe	SR	SR + RT	16 Months	8 Months to Remission	--
Ikawa et al. [34]	48/F	L Elbow	R Cerebellum	SR + RT + CH	SR + RT + CH	60 Months	13 Months	73 Months
Eggers et al. [35]	69/M	R Inguinal Area	R Pons + Midbrain + Leptomeninges	SR + RT + CH	RT	14 Months	2 Months	16 Months
Barkdull et al. [36]	55/M	R Frontal Scalp	Calvaria + Dura	--	SR + RT + CH	Present at Dx	10 Months	10 Months
Faye et al. [37]	85/M	R Temporal Region	R Temporal + L Frontal + Meninges	SR + RT	CH	12 Months	--	--
	68/M	--	R Posterior Parietal and Leptomeninges	SR	SR + RT	12 Months	17 Months	29 Months
Chang et al. [38]	45/M	L Temporal Skin	Cavernous Sinuses + L Trigeminal Cistern + Internal Acoustic Meatus	SR + RT + CH	RT + CH	24 Months	≥3 Months	--
Feletti et al. [39]	65/F	--	Pituitary	SR	SR + SRS + CH	42 Months	≥8 Months	--
De Cicco et al. [40]	69/M	--	Brain	SR + RT + CH	--	24 Months	4 Months	28 Months
Bailey et al. [41]	75/F	L Nasal Ala	R Parietal	SR + RT	SR + RT + G	17 Months	7 Months	24 Months
	77/F	L Frontoparietal Scalp	L Parietal	--	SR	Present at Dx	≥16 Months	--
	51/F	R Calf	R Posterior Temporal Lobe	SR	SR + RT + G	48 Months	≥21 Months to Remission	--
Abul-Kasim et al. [42]	65/M	--	R Parietal Parasagittal + Cerebellar Leptomeninges	RT	SRS + RT	--	8 Months	8 Months
Seaman et al. [43]	78/M	--	L Cerebellopontine Angle	SR	SR + RT + CH	2 Months	7 Months to Remission	--
Ho et al. [44]	60/M	L Shoulder	Clivus + Cavernous Sinus	SR + RT	RT	4 Months	≥6 Months	--
Ramachandran et al. [45]	51/M	L Frontoparietal Scalp	Bilateral Frontal + Bilateral Cerebral Masses	--	RT + CH + IM	--	1 Week	1 Week
Caramanti et al. [46]	56/M	--	L Frontal Lobe	--	SR + RT + CH	0 Months	3 Months	3 Months
Kwon et al. [47]	65/F	--	Brain	--	IM	--	19 Months to Remission	--
Grubb and Hankollari [48]	71/M	L Frontoparietal Scalp	Bilateral Frontal + Bilateral Cerebral Masses	--	RT + CH + IM	--	1 Week	1 Week
Ansgar et al. [49]	60/M	R Lateral Forearm + Hand	Bihemispheric + Fronto-Basal + Parietal + Supra-Tentorial + Occipital	RT + CH + IM	RT + IM	23 Months	11 Months	33 Months
Rizzo et al. [50]	48/M	--	Brain	CH + IM	SRS + IM	2 Months	7 Months	9 Months
Fife et al. [51]	70/F	--	Brain	CH + IM	SRS + IM	2 Months	7 Months	9 Months
	48/F	L Thigh	Right Occipital	CH	IM	12 Months	2.5 Months	14.5 Months
	67/F	--	Meningeal	CH	SRS + CH + IM	6 Months	11 Months	17 Months
	66/M	--	R Medial Temporal + L Occipital Lobe	SR + CH	SRS + IM	14 Months	39 Months to Remission	--
Yu et al. [52]	69/F	--	L Cerebellar	SR + RT + CH + IM	SRS + RT + IM	21 Months	≥39 Months	--
This report	87/M	R Cheek	R Frontal Lobe	SR + RT	SR + SRS	24 Months	≥9 Months to Remission	--

The diagnosis of MCC may start with cutaneous tumor recognition. The early identification of the neoplasm is important as this rare form of cancer has aggressive tendencies to invade surrounding tissue, travel to nearby lymph nodes, and have hematogenous spread to metastasize. Based on a prospective study done on 5,832 patients, the common anatomic site of the primary lesion was found to be the head and neck, followed by the upper extremities, including the shoulder, and lastly the lower extremities [52,53]. Based on our review, 61% of the cases reported a primary lesion with 65% of lesions located in the head and neck region, followed by 16% in the upper extremities and 16% in the lower extremities, showing a similar pattern found in the study described above. The other cases either did not report the primary lesion or were unable to identify one. Regardless, the inability to locate the primary site of the tumor is found to have survival benefits for the patient and has been attributed to immune-mediated mechanisms that eliminate the initial growth [54]. In our review, we found the mean survival time of patients that presented with a primary lesion and had overall survival time reported (61% of cases) to be 20 months (range: 0.25-73 months) compared to 15 months in patients that did not present with a primary lesion and had reported survival time (14% of cases). This suggests that patients without a primary lesion may have a slight benefit due to the immune-mediated attack on MCC, resulting in an increase in survivability. Following the initial lesion, the metastatic spread was found in 7.9% of patients diagnosed with MCC, with the neurometastatic involvement of MCC accounting for less than 1%. The low rate of CNS metastasis has made a diagnosis of brain involvement challenging as well; a neuroanatomic propensity of metastatic invasion by MCC has not been reported. In this review, we attempt to analyze each case of neurometastatic MCC. However, we were unable to identify a pattern of spread. Instead, we found it to have varying effects on the CNS as is described in Table 1.

Following tumor identification, histological analysis reveals both epithelial and neuroendocrine characteristics; therefore, immunostaining is needed to definitively distinguish MCC from other malignancies such as melanoma, cutaneous small cell carcinoma, and lymphoma. Several studies proposed characteristic stains for MCC to be positive for epithelial markers such as cytokeratin (CK), CAM 5.2, AE1/AE3, and neuroendocrine markers neuron-specific enolase (NSE), synaptophysin, and chromogranin A (CgA). Negative markers of vimentin, melan-A, S-100, melanoma-specific antigen, and Human Melanoma Black 45 (HMB-45) rule out melanoma, leukocyte common antigen (LCA) with lymphoma, and thyroid transcription factor 1 (TTF-1) with small cell carcinoma [55,56]. In our review, 53% of the cases reported immunohistochemical staining characteristics of MCC. Of the results, CK20 was the most consistently positive marker, found in 63% of reported cases (17 of 27 cases), followed by NSE, synaptophysin, and CgA, which were positive in 44%, 33%, and 30%, respectively (Table 2). Less frequently, cluster of differentiation (CD) 56, AE1/AE3, CAM 5.2, and cytokeratin (unspecified) were found to be positive in several cases. Clinically important negative markers such as LCA and TTF-1 were described in 22% of the tested cases. Melanoma-signifying stains such as S-100, vimentin, desmin, melanoma-specific antigen, and HMB45 were found to be negative in 22% of the cases pooled. Based on the results of our data, we are in congruence with the guidelines and recommend CK20, NSE, synaptophysin, and CgA to be used as positive markers to aid in the diagnosis of MCC with CNS involvement.

**Table 2:** Immunohistochemical characteristics of 27 cases of MCC and brain metastasis MCC, Merkel cell carcinoma; HMB-45, Human Melanoma Black 45; CK20, cytokeratin 20; CD56, cluster of differentiation 56

Immunohistochemistry Marker	Positive	Negative
Neuron-Specific Enolase (NSE)	12	1
CK20	17	0
Chromogranin A (CgA)	8	1
Leukocyte Common Antigen (LCA)	1	6
Epithelial Membrane Antigen (EMA)	1	0
Vimentin	1	1
Desmin	0	1
Synaptophysin	9	0
AE1/AE3	3	0
Calcitonin	1	1
CAM 5.2	3	0
S-100	0	2
Melanoma-Specific Antigen (MSA)	0	1
Cytokeratin (Unspecified)	3	1
Thyroid Transcription Factor 1 (TTF-1)	0	6
HMB45	0	1
CD56	4	1

An additional diagnostic test has been developed since Merkel cell polyomavirus (MCPyV) was identified in tumor tissue and found as a risk factor for developing MCC by interacting with p53 and retinoblastoma protein (RB) [57]. Of the 114 patients analyzed with MCC, it was found that 91 (79.8%) cases presented with MCPyV, concluding the frequent prevalence of the virus in the tumor [58]. Furthermore, the study found that patients that tested positive for the virus had better prognoses compared to the MCPyV-negative patients with MCC. In our study, 18 cases of MCC and brain metastasis were reported after the identification of MCPyV. Of those reported, 28% reported testing for the virus with 100% being negative. Due to the lack of information regarding the virus in the other patients, we were unable to compare the prognostic effects of MCPyV.

Once a diagnosis of MCC has been identified, the primary lesion is often treated surgically if the tumor is in an accessible location and the patient is a surgical candidate. Adjuvant radiation therapy (50 Gy) often follows and is paired with chemotherapy in the case of extranodal disease. If surgery is not indicated, radiation therapy is the primary modality chosen [55]. However, a randomized control study found that radiation therapy had no measurable benefits in survival and the primary benefit may only be attributed to symptom control [59]. In the case of MCC brain metastasis, no curative treatment has been found, and chemotherapy continues to be the main regimen for this stage of the disease with little evidence of benefit. A retrospective study found a high response rate to the chemotherapeutic treatment of metastatic MCC but a median progression-free survival time to be three months, suggesting chemoresistant tumors [60]. With the inability to effectively treat metastatic MCC, the median survival time has been shown to be 10 months [61]. In our review, we were able to deduce overall survival time in 24 cases with the remaining 28 lacking sufficient information. The most reported treatment plan for brain metastasis of MCC was surgery (surgical resection {SR}) combined with adjuvant radiation therapy (RT) and chemotherapy (CH) (28% of cases) with an average survival time of 22.3 months (range: 3-70 months). Followed by RT combined with CH (15% of cases), we found a mean survival time of 13.2 months (range: 9.5-18 months) with 16% showing complete remission (Table 1). Our data shows survival times being less than two years when using chemoradiation therapy; however, due to the small sample size, there were large deviations in survivability. More recently, immunotherapeutic medications have been created to treat MCC since the finding of programmed cell death protein 1 (PD-1) on tumor-infiltrating lymphocytes and programmed death-ligand 1 (PD-L1) on tumor cells. Several studies conducted found PD-1 and PD-L1 inhibitors to have durable responses and an increase in progression-free survival times. In our review, we found nine patients after 2019 treated with immunotherapy for MCC and brain metastasis often in combination with other regimens. Of the nine cases, three (33%) were in complete remission, and the other six had a mean survival time of 14.75 months (range: 0.25-33 months).

In the case of the 87-year-old male we reported, the patient had a complete response to a PD-1 inhibitor (pembrolizumab) in combination with stereotactic radiosurgery with a progression-free survival time of five months. Conclusions of immunotherapy efficacy cannot be made based on the small sample size; however, the data that we were able to analyze shows promising responses to immunotherapy and its ability to treat MCC metastatic disease in conjunction with chemoradiation therapy.

## Conclusions

In summary, neurometastatic MCC is a rare and aggressive malignancy that is associated with high mortality. Our review revealed that the detection of the tumor may prove to be difficult as the primary lesion is not always identifiable; however, if found, it is likely to be in the head and neck region. The immunohistochemical staining of the tumor or lymph node is always indicated to aid in the diagnosis of the disease and is often associated with positive epithelial and neuroendocrine markers such as CK20, NSE, synaptophysin, and CgA. Lastly, previous therapeutic regimens consist of chemoradiation therapy that has not proven to be successful in treating the disease. New immunotherapeutic medications for MCC and brain metastasis show potential for improving response and survivability. Future studies are needed to determine its efficacy for use in this population.
